# Efficacy of Traditional Chinese Medicine on Animal Model of IgA Nephropathy: A Systematic Review and Meta-Analysis

**DOI:** 10.1155/2022/6106993

**Published:** 2022-12-26

**Authors:** Tian-ying Chang, Hong-an Wang, Yin-ping Wang, Jin-hui Ma, Di Zou, Shou-Lin Zhang, Lehana Thabane

**Affiliations:** ^1^EBM Department, The Affiliated Hospital to Changchun University of Chinese Medicine, Changchun 130021, China; ^2^Nephrology Department, The Affiliated Hospital to Changchun University of Chinese Medicine, Changchun 130021, China; ^3^Department of Health Research, Evidence, and Impact, McMaster University, Hamilton, ON, Canada; ^4^Biostatistics Unit, St Joseph's Healthcare-Hamilton, Hamilton, ON, Canada

## Abstract

**Objective:**

Traditional Chinese medicine (TCM) has a long history in the treatment of Immunoglobulin A nephropathy (IgAN). A large number of animal experiments focused on the TCM treatment of IgAN are conducted every year. The evidence for these preclinical studies is not clear. This study summarized and evaluated the results of animal experiments on TCM treatment for IgAN.

**Methods:**

We systematically searched animal studies from 6 databases from inception to August 30, 2022. We included Chinese studies from the key magazine of China technology. The quality of the included studies was evaluated with the SYRCLE animal experimental bias risk assessment tool and the Grading of Recommendations Assessment, Development, and Evaluation (GRADE).

**Results:**

Out of 832 records identified in the initial search, 30 studies were selected. The results indicated that, compared with the control group, the TCM treatment group improved 24 h urine protein (24 h-UP) level (standardized mean difference (SMD) 3.57, 95% confidence interval (CI) 4.48 to 2.66, *P* < 0.001), urine red blood cell (U-RBC) (SMD 13.66, 95% CI 17.99 to 9.32, *P* < 0.001), serum creatinine (Scr) (mean difference (MD) 10.89, 95% CI 17.00 to 4.77, *P* < 0.001), blood urea nitrogen (BUN) (MD 2.44, 95% CI 3.42 to 1.47, *P* < 0.001), tumor necrosis factor-*α* (TNF-*α*) (MD 171.28 to 95% CI 323.68 to 18.88, *P*=0.03), transforming growth factor-*β*1 (TGF-*β*) (SMD 4.02, 95% CI 7.26 to 0.77, *P*=0.02), matrix metalloproteinase-9/tissue inhibitors of metalloproteinase-1(MMP-9/TIMP-1) (MD 0.03, 95% CI 0.00 to 0.06, *P*=0.02), nephrin mRNA (SMD 3.39, 95% CI 2.59 to 4.18, *P* < 0.001). However, there is no difference in albumin level (MD 1.10, 95% CI 0.06 to 2.26, *P*=0.06) and interleukin-6 (IL-6) (MD 170.77, 95% CI 365.3 to 23.75, *P*=0.09).

**Conclusions:**

TCM can improve 24 h-UP, U-RBC, Scr, BUN, MMP-9/TIMP-1, TNF-*α*, TGF-*β*, and nephrin mRNA of IgAN animal models. Moreover, there is a need for rigorous reporting of preclinical research methodology, which is essential to support the quality of preclinical research. *Registration*. This review was registered with a systematic review record CRD42020171404 in the PROSPERO database.

## 1. Introduction

IgA nephropathy (IgAN) refers to primary glomerulonephritis with a large number of IgA or IgA-based immune complex granules deposited in the mesangial area. It is the most common primary glomerulonephritis in the world at present. [1] The modern medical treatment for IgAN is limited [2, 3]; Chinese medicine (including herb decoctions under the guidance of TCM theory, extract of herbs or decoctions, and patents) has been widely approved for its positive role in the prevention and treatment of IgAN [4].

The incidence of primary glomerular disease accounts for 30% in Asia, and it is also one of the leading causes of end-stage renal disease (ESRD) in China. Data show that the incidence of this disease has been gradually increasing in recent years. [5] The prognosis of IgAN is not optimistic. After treatment, about 50% of patients still progress to ESRD within 30 years. [6] Patients with ESRD can only rely on kidney transplantation or dialysis to maintain their lives, which puts a massive psychological and economic burden on individuals, families, and society. Kidney function preservation and remission of proteinuria are the principles for its treatment. [7] Glucocorticoids and immunosuppressants can reduce urinary protein levels in IgAN patients and improve the prognosis. [3] However, severe adverse reactions, insensitivity, and drug resistance occur sometimes. TCM has certain advantages in the treatment of IgAN. It could delay the development of IgAN in the early stage, synergistically treat IgAN with immunosuppressants and glucocorticoids in the middle stage, and improve life quality in the end stage. [8] However, the results of these studies were not systemically evaluated. Systematic evaluation of animal experiments has become a new trend and an essential means to integrate animal experimental results, improve the quality of animal experiments, and guide clinical research. It is also an important channel to connect basic research and clinical trials. To further clarify the mechanism of Chinese medicine in the treatment of IgAN, this study intends to use the meta-analysis method to systematically evaluate the intervention of TCM in experimental IgAN animal models, to expand further the evidence of the mechanism of TCM in the treatment of IgAN, and to provide reference data for follow-up research.

## 2. Materials and Methods

This review was registered with a systematic review record CRD42020171404 in the PROSPERO database.

### 2.1. Search Strategy

Databases including PubMed, Cochrane Library, Embase, CNKI, VIP, and Wanfang data were searched for the literature from the inception to 4 April 2020 without language restriction. MeSH terms and keywords (“Immunoglobulin A Nephropathy” OR “Immunoglobulin A”) AND (“Chinese Medicine” OR “Chinese Herbal Medicine”) AND (“Animal” OR “Rat” OR “Mice”) were used to search studies.

### 2.2. Inclusion and Exclusion Criteria

#### 2.2.1. Eligibility Criteria

(1) Participants: models of IgAN (rats or mice); (2) intervention(TCM herb decoctions, extract, and patents): with all doses and durations; (3) control: distilled water treated, saline water treated, and same solvent or no treatment; (4) outcomes: 24 h-UP, U-RBC, Scr, BUN, Alb, MMP-9/TIMP-1, IL-6, TNF-*α*, TGF-*β*, and nephrin mRNA; (5) Study design: randomized controlled research studies.

#### 2.2.2. Exclusion Criteria

(1) Participants: *in vitro* studies and research in humans; (2) intervention: TCM without dose unit or TCM was not given by oral gavage administration; (3) control: other TCM; (4) study design: case reports, crossover studies, and studies without a separate control group; (5) pilot studies; (6) reviews; (7) conference papers; (8) studies without full text. If studies were repeatedly published, we chose the one with the largest sample size.

### 2.3. Data Extraction

Two reviewers (TYC and HAW) independently extracted the following information according to the predesigned file from selected studies: characteristics of studies (first author, publication year, TCM treatment composition, type of models, modeling method; individual data for each study, including animal number, species, gender, weight, treatment time, and mode of administration). The outcome measurement includes 24 h-UP, U-RBC, Scr, BUN, Alb, MMP-9/TIMP-1, IL-6, TNF-*α*, TGF-*β*, and nephrin mRNA. If outcomes were presented at different time points, variables were extracted from the last time point. If studies had more than one experiment group sharing one control group, the control group would be separated into multiple groups (the number was the same as the experiment groups), incorporating these comparisons into this meta-analysis. Any disagreements regarding extracted data were resolved through discussion, if necessary, by a third party (SLZ).

### 2.4. Quality Assessment

The included studies were evaluated with SYRCLE's Risk of Bias Tool for Animal Studies (8). Ten items were used to analyze the methodological quality: (1) sequence generation; (2) baseline characteristics; (3) allocation concealment; (4) random housing; (5) blinding (performance bias); (6) random outcome; (7) blinding (detection bias); (8) incomplete outcome data; (9) selective outcome reporting; (10) other sources of bias. Two reviewers (TYC and HAW) assessed the risk of bias independently, and if necessary, a third party (SLZ) adjudicated the judgment.

### 2.5. Statistical Analysis

We used RevMan version 5.3 by the Cochrane Collaboration Network to analyze the data. Continuous data were expressed as the mean difference with a 95% confidence interval (CI). Categorical data were calculated with the risk ratio (RR) and 95% CI. We evaluated heterogeneity with the Higgins *I*^2^ test. If *I*^2^ > 50%, a random-effect model was used for meta-analyses; if not, a fixed-effect model was used. Publication bias was assessed by a funnel plot. The quality of evidence was rated with the Grading of Recommendations Assessment, Development, and Evaluation (GRADE) [9].

## 3. Results

### 3.1. Study Inclusion

**Figure 1:**
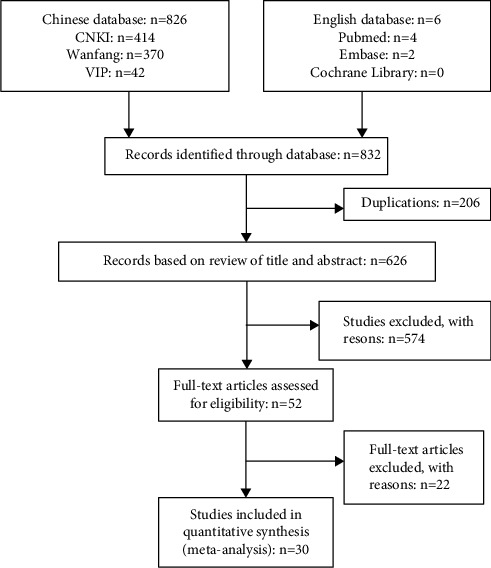
Literature screening process.

In the initial search, 832 related studies were obtained and 206 repetitive studies were excluded as duplications by EndNote X9 software. The rest studies were further screened by reviewing titles, abstracts, and the list of Chinese Core Journals. The rest 52 studies were full-text screened, and 19 studies were excluded by a nonunified treatment dose. At last, 30 eligible studies were identified. All the literature was published in Chinese, and all trials were conducted in China. The literature screening process and results are shown in Figure 1.

### 3.2. Study Characteristics

The 30 included studies involved 681 animals, the treatment group (*n* = 340), and the control group (*n* = 341). And among these animals, 150 were Wistar rats, 511 were Sprague Dawley rats, and 20 were BALB/C mice. 17 studies [10–26] used male rats, five studies [27–32] used female rats, four studies [32–35] used half male and half female, and 4 studies [16, 33, 36, 37] did not describe. The animal model of IgA nephropathy was established by bovine serum albumin (BSA) + lipopolysaccharide (LPS) + carbon tetrachloride (CCL4) in 22 studies [10, 11, 13–22, 24–26, 32–39], and BSA + staphylococcal enterotoxin-B (SEB) in 8 studies [12, 23, 27–31, 40]. 19 studies [10, 12–15, 17, 18, 21–24, 27, 29, 30, 32, 35, 37–39] used 24 h-UP as a measurement result, 10 studies [11–13, 15, 17, 20, 22, 24, 27, 29] used U-RBC, 15 studies [12, 13, 15, 16, 18, 21, 24, 27, 29, 30, 32, 35, 37–40] used Scr, 12 studies [12, 13, 15, 16, 18, 21, 24, 29, 30, 35, 37, 39, 40] used BUN, six studies [15, 24, 30, 32, 35, 38] used Alb, two studies [14, 38] used nephrin mRNA, two studies [13, 33] used TGF-*β*1, two studies [11, 14] used MMP9/TIMP-1, three studies [12, 35, 36] used IL-6, and TNF-*α* was used in 3 studies [18, 20, 36], and these characteristics are shown in Table 1. The quality of evidence was rated with GRADE.

### 3.3. Methodological Quality of Included Studies

Sequence generation was mentioned in 7 studies. [13, 14, 21, 33, 35, 38, 39] Incomplete outcome data were mentioned in 6 studies. [16, 20, 22, 25, 30, 36] All studies mentioned selective outcome reporting and other sources of bias. All studies did not mention baseline characteristics, allocation concealment, random hosing, blinding (performance bias), random outcome assessment, and blinding (detection bias). The methodological quality of the included studies is shown in Table 2.

### 3.4. 24-Hour Urinary Protein

**Figure 2:**
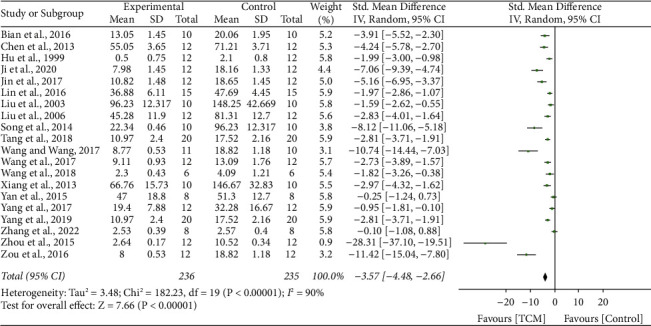
Forrest plot of TCM vs. control for the outcome of 24 h urinary protein.

20 studies [10–15, 17, 18, 21–24, 27, 29, 30, 32, 35, 37–39] reported the impact of TCM on 24 h-UP, and results showed significant heterogeneity (*I*^2^ = 90%, *P* < 0.001). A random-effect model was conducted. The meta-analysis revealed that the 24-hour urine protein level was significantly improved compared with the control group treatments (SMD 3.57, 95% CI 4.48 to 2.66, *P* < 0.001) (Figure 2).

### 3.5. Urinary Red Blood Cell

**Figure 3:**
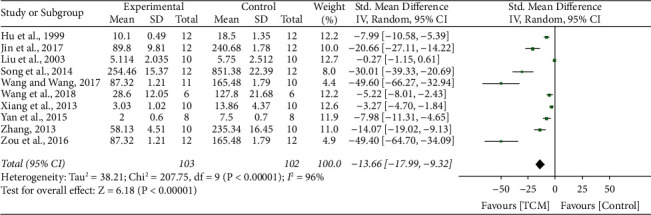
Forrest plot of TCM vs. control for the outcome of urinary red blood cell.

10 studies [11–13, 15, 17, 20, 22, 24, 27, 29] reported the impact of TCM on U-RBC, and results showed significant heterogeneity (*I*^2^ = 96%, *P* < 0.001). A random-effect model was conducted. The meta-analysis revealed that the U-RBC level was significantly improved compared with the control group treatments (SMD 13.66, 95% CI 17.99 to 9.32, *P* < 0.001) (Figure 3).

### 3.6. Serum Creatinine

**Figure 4:**
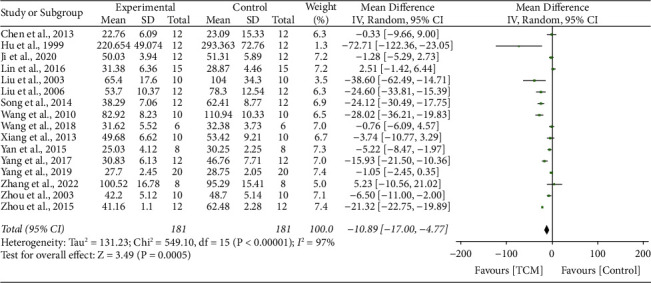
Forrest plot of TCM vs. control for the outcome of serum creatinine.

15 studies [12, 13, 15, 16, 18, 21, 24, 27, 29, 30, 32, 35, 37–40] reported the impact of TCM on Scr, and results showed significant heterogeneity (*I*^2^ = 97% *P* < 0.001). A random-effect model was conducted. The meta-analysis revealed that the Scr level was significantly improved compared with the control group treatments (MD 10.89, 95% CI 17.00 to 4.77, *P* < 0.001) (Figure 4).

### 3.7. Blood Urea Nitrogen

**Figure 5:**
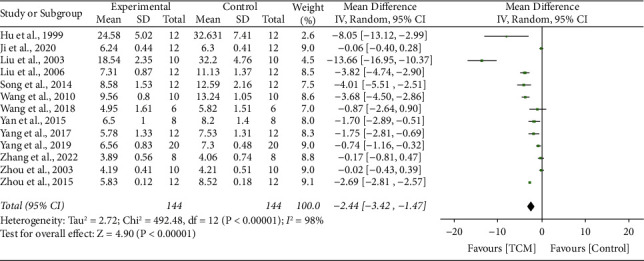
Forrest plot of TCM vs. control for the outcome of blood urea nitrogen.

13 studies [12, 13, 15, 16, 18, 21, 24, 27, 29, 30, 32, 35, 37–40] reported the impact of TCM on BUN, and results showed significant heterogeneity (*I*^2^ = 98%, *P* < 0.001). A random-effect model was conducted. The meta-analysis revealed that the BUN level was significantly improved compared with the control group treatments (MD 2.44, 95% CI 3.42 to 1.47, *P* < 0.001) (Figure 5).

### 3.8. Albumin

**Figure 6:**
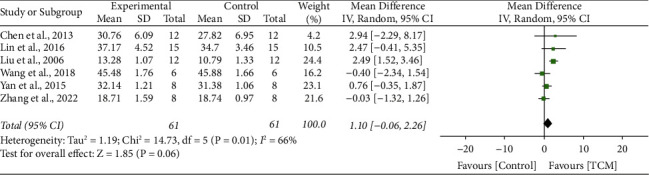
Forrest plot of TCM vs. control for the outcome of albumin.

6 studies [15, 24, 30, 32, 35, 38] reported the impact of TCM on Alb, and results showed significant heterogeneity (*I*^2^ = 66%, *P*=0.01). A random-effect model was conducted. The meta-analysis revealed no difference between the two groups in improving Alb level (MD 1.10, 95% CI 0.06 to 2.26, *P*=0.06) (Figure 6).

### 3.9. MMP 9/TIMP-1

**Figure 7:**
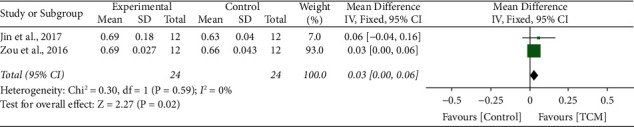
Forrest plot of TCM vs. control for the outcome of MMP-9/TIMP-1.

2 studies [11, 22] reported the impact of TCM on MMP-9/TIMP-1, and no significant heterogeneity was tested between these two studies (*I*^2^ = 0%, *P*=0.59, *P*=0.59). A fixed-effect model was conducted. The meta-analysis revealed that the MMP-9/TIMP-1 level was significantly improved compared with the control group treatments (MD 0.03, 95% CI 0.00 to 0.06, *P*=0.02) (Figure 7).

### 3.10. Interleukin-6

**Figure 8:**
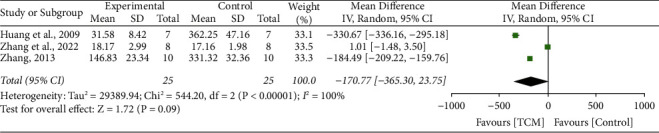
Forrest plot of TCM vs. control for the outcome of IL-6.

3 studies [20, 36] reported the impact of TCM on IL-6, and no significant heterogeneity was tested between these two studies (*I*^2^ = 100%, *P* < 0.001). A random-effect model was conducted. The meta-analysis revealed no difference between the two groups in improving IL-6 level (MD 170.77, 95% CI 365.3 to 23.75, *P*=0.09) (Figure 8).

### 3.11. Tumor Necrosis Factor-*α*

**Figure 9:**
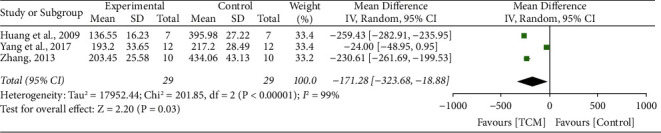
Forrest plot of TCM vs. control for the outcome of tumor necrosis factor-*α*.

Three studies [18, 20, 36] reported the impact of TCM on TNF-*α*, and no significant heterogeneity was tested between these two studies (*I*^2^ = 99%, *P* < 0.001). A random-effect model was conducted. The meta-analysis revealed that the TNF-*α* level was significantly improved compared with the control group treatments (MD 171.28 to 95% CI 323.68 to 18.88, *P*=0.03) (Figure 9).

### 3.12. Transforming Growth Factor-*β*1

**Figure 10:**
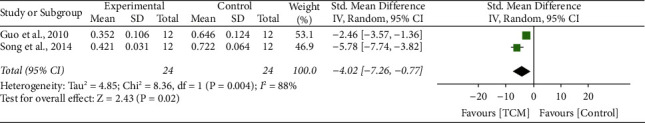
Forrest plot of TCM vs. control for the outcome of transforming growth factor-*β*1.

Two studies [13, 33] reported the impact of TCM on TGF-*β*1, and no significant heterogeneity was tested between these two studies (*I*^2^ = 88%, *P*=0.004). A fixed-effect model was conducted. The meta-analysis revealed that the TGF-*β*1 level was significantly improved compared with the control group treatments (SMD 4.02, 95% CI 7.26 to 0.77, *P*=0.02) (Figure 10).

### 3.13. Nephrin mRNA

**Figure 11:**
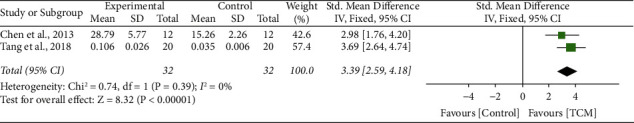
Forrest plot of TCM vs. control for the outcome of nephrin mRNA.

2 studies [14, 38] reported the impact of TCM on nephrin mRNA, and no significant heterogeneity was tested between these two studies (*I*^2^ = 98%, *P*=0.39). A fixed-effect model was conducted. The meta-analysis revealed that the nephrin mRNA level was significantly improved compared with the control group treatments (SMD 3.39, 95% CI 2.59 to 4.18, *P* < 0.001) (Figure 11).

### 3.14. Subgroup Analyses

**Figure 12:**
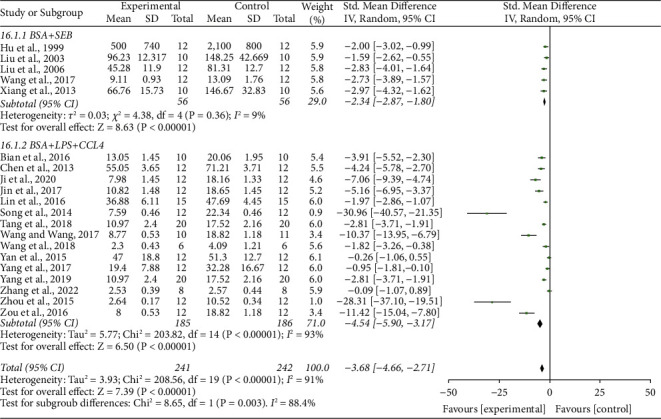
Forrest plot of TCM vs. control for subgroup outcome of 24-hour urinary protein.

We conducted a subgroup analysis by different modeling methods. The TCM can significantly reduce 24 h-UP on the BSA + LPS + CCL4 model (SMD 4.54, 95% CI-5.90 to 3.17, *P* < 0.001) and the BSA + SEB model (SMD-2.34, 95% CI-2.87 to 1.80, *P* < 0.001), and the overall effect is (SMD 3.68, 95% CI 4.66 to 2.71, *P* < 0.001) (Figure 12).

### 3.15. Publication Bias Test

**Figure 13:**
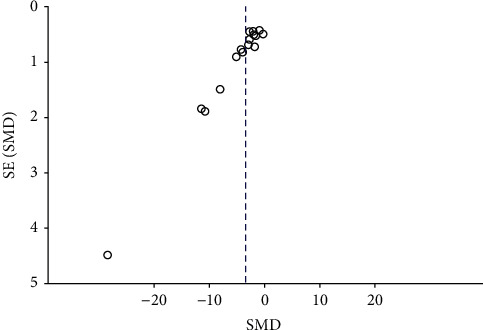
Funnel plot of the SMD in 24 h-UP.

We conducted a funnel plot to indicate publication bias and potential publication bias observed in Figure 13.

### 3.16. Sensitivity Analysis

Significant heterogeneity was found in the meta-analysis of 24 h-UP, U-RBC, Scr, BUN, albumin, IL-6, TNF-*α*, and TGF-*β*1. We conducted a series of sensitivity tests by excluding better-designed studies to examine the robustness of our results, and we found that the results were consistent.

## 4. Discussion

The renal protective effect of TCM has been extensively researched in different animal experimental models, and our systematic review and meta-analysis intend to analyze whether TCM treatment exerted an effect on IgAN animal models. The results indicated that oral gavage TCM could significantly lower levels of 24 h-UP, U-RBC, Scr, BUN, albumin, MMP-9/TIMP-1, IL-6, TNF-*α*, TGF-*β*1, and nephrin mRNA.

Twenty-one TCM treatments were used in our systematic review, eleven were decoctions, and eight were patent. The main medicinal ingredients of TCM are the following: Rehmannia, Astragalus, Chinese yam, Puhuang, Imperata root, Poria, Alisma, Chinese wolfberry, and Rhubarb. These TCM were taken under the guidance of Nourishing Yin and Qi, tonifying kidney, and hemostasia methods.

IgAN, as primary glomerulonephritis, first identified by Berger and Hinglais in 1968, represents the leading cause of kidney failure among East Asian populations. [41] Aberrant glycosylation of IgA1 elicits an autoimmune response, generating antiglycan antibodies. [42] Consequent immune complexes deposit in the glomerular mesangium, which activates the complement pathway, stimulates mesangial cells, and induces the secretion of cytokines, finally resulting in inflammation and fibrosis. IgAN is an autoimmune disease wherein immune complexes cause renal injury. [43] Studies of animal experiments showed that urinary protein could induce renal tubular epithelial cell damage, so urinary protein has been used as an independent factor in evaluating renal prognosis. [44, 45].

The pathogenesis of IgAN includes environmental factors, genetic factors, and immune factors, among which immune factors have been the primary targets for the study of the treatment of the disease. At present, the cytokines that play an essential role in the pathogenesis and progression of IgAN are IL-6, TNF-*α*, TGF-*β*1, and MMP-9/TIMP-1. TNF-*α* is produced by activated monocytes; under normal conditions, an appropriate amount of TNF-*α* has a protective effect on the body, and excessive TNF-*α* causes immune damage to the body. Studies have shown that mononuclear macrophages infiltrate into renal tissue to release TNF-*α*, resulting in focal glomerular damage, resulting in gross hematuria, [46, 47] consistent with clinical studies. [47, 48] IL-6 can induce B lymphocytes to differentiate and produce immunoglobulin, stimulate the proliferation of glomerular mesangial cells, and produce an extracellular matrix, thus increasing the burden on the kidney and leading to glomerular fibrosis. [49] Glomerulosclerosis is a common pathological feature of most immune and nonimmune renal diseases, and TGF-*β* has been recognized as the target of glomerular sclerotherapy. [50] TGF-*β* can promote the proliferation of mesangial cells and promote the synthesis and deposition of extracellular matrix and can stimulate glomerulosclerosis. [51] In the animal model of IgAN and patients with IgAN, the level of TGF-*β* in plasma and renal tissue increased. [52] TIMP-1 and MMP-9 form an MMP-TIMP complex at 1 : 1, blocking the binding of MMP-9 to the substrate, thus affecting the balance of ECM accumulation and degradation. [53, 54] Any imbalance between TIMP-1/MMP9 and ECM may lead to abnormal accumulation of ECM, glomerular disease, glomerular remodeling, hematuria, and proteinuria. The meta-analysis results showed that after TCM treatment, TNF-*α* and TGF-*β* could be reduced, and serum MMP-9/TIMP-1 content could be increased; the difference was statistically significant.

In addition, the study found that there is also podocyte destruction in the occurrence and development of IgAN. [55] Podocyte injury, especially the role of changes in the fissure diaphragm in the occurrence of IgAN proteinuria, has also attracted people's attention. Clinical studies have confirmed that the urinary protein level exceeding 1.0 g/d during renal biopsy indicates that the prognosis of patients is poor. [56, 57] Therefore, stabilizing the podocyte fissure diaphragm will be an important starting point for treating IgAN proteinuria. Nephrin, a sign of mature podocytes, is the first transmembrane protein found on the glomerular filtration barrier of SD, and it is also the “main body” part of SD. The nephrin deficiency in humans and rats will lead to classic SD deficiency and massive proteinuria. The results of the meta-analysis showed that the TCM treatment could significantly reduce the level of urinary protein and downregulate the expression of nephrin mRNA in podocytes in rats with IgA nephropathy, thus playing a role in the treatment of IgAN.

Plenty of animal experiments focused on TCM treatment for IgAN animal models have been conducted for years. For better quality of included studies, we chose studies from the list of Chinese Core Journals if published in Chinese. Animal models for IgAN were established in three kinds of rats/mice. Six studies constructed animal models with Wistar rats, one study constructed an animal model with BALB/C mice, and twenty-three studies used SD rats. Most studies constructed models with two methods. One method is oral gavage with BSA 400 mg/kg every other day, subcutaneous injection of CCL4 0.1 ml/weekly and benne oil 0.5 mL/weekly for nine weeks, and intravenous injection of LPS 0.05 mg at the sixth or eighth week. The other method is intravenous injection of SEB combined with oral gavage with BSA. IgAN animal experiments conducted outside China usually construct an animal model with a ddY mouse [58] which has an abnormally high concentration of IgA from 10 to 60 weeks, but rare hematuria. The BSA + SEB method usually constructs models with a high mortality rate after modeling. Most included studies used the BSA + LPS + CCL4 method to construct an animal model.

Methodological quality was low among animal experiments. We used GRADE to evaluate the certainty of the main outcomes of this meta-analysis, and all certainty is very low. Insufficient reporting of how these studies were conducted lowers the quality and increases the biases. Six of ten SYRCLE's tool items were reported as unclear in all included studies, which means researchers need more training in methodological ability. Evidence-based medicine ability should be emphasized to TCM researchers and practitioners. The animal modeling method is also a source of heterogeneity. Furthermore, our results were consistent with the sensitivity of excluding studies with different modeling methods.

Through this study, we can find that although there are a large number of IgAN animal experimental studies published, most of them are not fully reported in the methods of model establishment, animal feeding, and model evaluation. High-quality systematic evaluation of animal experiments will help to prevent the waste of laboratory animals and test participants by carrying out unnecessary, ineffective, or less information research [59]. Therefore, a high-quality original research design is essential to provide research evidence for treating IgAN with traditional Chinese medicine.

This study showed that TCM intervention in IgAN animals could reduce levels of 24 h-UP, U-RBC, Scr, BUN, TNF-*α* and upregulate the contents of serum TGF-*β*1, nephrin mRNA, and MMP-9/TIMP-1. According to different animal species and modeling methods in the subgroup analysis, the results show that TCM significantly affects IgAN SD and Wistar rats; the results are statistically significant. This meta-analysis reinforces the evidence that TCM has a protective effect in experimental IgAN animal models. However, the methodological quality of these studies needs to be improved.

## Figures and Tables

**Table 1:** Basic characteristics of the included studies.

Study	Species	Number (T/C)	Gender m/f	Weight (g)	Model method	Interventions		Outcome
						Treatment	Control	
Bian et al., 2016	SD rats	10/10	0/40	150 ± 10	BSA + LPS + CCL4	Decoction	Distilled water	1
Chen et al., 2013	SD rats	12/12	30/30	200 ± 20	BSA + LPS + CCL4	Decoction	Saline	1,3,5,10,19,20,21
Guo et al., 2010	SD rats	12/12	NR	220–260	BSA + LPS + CCL4	Patent	Distilled water	6,22
Huang et al., 2009	SD rats	7/7	NR	145–155	BSA + LPS + CCL4	Patent	Saline	8,9
Jin et al., 2017	Wistar rats	12/12	0/80	200–220	BSA + LPS + CCL4	Patent	Distilled water	1,2,7
Liu et al., 2003	SD rats	10/10	0/50	180 ± 10	BSA + SEB	Patent	Distilled water	1,2,3,4,14
Song et al., 2014	Wistar rats	12/12	0/60	150 ± 10	BSA + LPS + CCL4	Decoction	Distilled water	1,2,3,4,6
Tang et al., 2018	SD rats	20/20	0/80	180 ± 20	BSA + LPS + CCL4	Decoction	Saline	1,10
Wang et al., 2018	SD rats	6/6	0/30	180–220	BSA + LPS + CCL4	Decoction	Saline	1,2,3,4,5,11,12,13
Wang et al., 2010	SD rats	10/10	NR	180–220	BSA + LPS + CCL4	Extract	Distilled water	3,4,23,24
Wang and wang, 2017	Wistar rats	10/11	0/80	180 ± 20	BSA + LPS + CCL4	Patent	Saline	1,2
Xiang et al., 2013	SD rats	10/10	60/0	200 ± 20	BSA + SEB	Patent	Saline	1,2,3
Yang et al., 2019	SD rats	20/20	NR	180 ± 20	BSA + LPS + CCL4	Decoction	Saline	1,3,4
Yang et al., 2017	SD rats	12/12	0/60	180 ± 20	BSA + LPS + CCL4	Decoction	Distilled water	1,3,4,9,26
Zhang et al., 2016	SD rats	12/12	0/72	180 ± 20	BSA + LPS + CCL4	Decoction	Saline	26
Zhang, 2013	Wistar rats	10/10	0/60	240–260	BSA + LPS + CCL4	Decoction	Distilled water	2,8,9
Zhou et al., 2015	SD rats	12/12	0/48	180–200	BSA + LPS + CCL4	Decoction	Distilled water	1,3,4
Zou et al., 2016	Wistar rats	12/12	0/72	160–200	BSA + LPS + CCL4	Patent	Distilled water	1,2,7
Zeng et al., 2004	SD rats	12/12	62/0	140 ± 10	BSA + SEB	Patent	Distilled water	36,37
Hu et al., 1999	SD rats	12/12	60/0	180 ± 10	BSA + SEB	Patent	Distilled water	1,2,3,4,27,28,29
Lin et al., 2016	SD rats	15/15	25/25	130–160	BSA + LPS + CCL4	Patent	Distilled water	1,3,5,14,15,27,30
Liu et al., 2006	SD rats	12/12	0/48	180 ± 10	BSA + SEB	Decoction	Tap water	1,3,4,5,
Sun et al., 2004	SD rats	12/12	60/0	140 ± 10	BSA + SEB	Patent	Distilled water	6
Wang et al., 2017	SD rats	12/12	0/36	200–210	BSA + SEB	Patent	Saline	1
Yan et al., 2015	SD rats	8/8	0/56	180 ± 10	BSA + LPS + CCL4	Extract	Saline	1,2,3,4,5,12,13
Ye et al., 2014	SD rats	10/10	0/60	180–220	BSA + LPS + CCL4	Decoction	Distilled water	31,32,33,34,35
Zhou et al., 2003	BALB/C mice	10/10	0/40	20	BSA + SEB	Decoction	Saline	3,4,23,24,27
Cao et al., 2009	SD rats	12/12	0/48	220–260	BSA + LPS + CCL4	Patent	Distilled water	16,17,18
Yu et al., 2013	SD rats	10/10	29/29	180–220	BSA + LPS + CCL4	Patent	Distilled water	25
Zhang et al., 2022	SD rats	8/8	8/8	200 ± 20	BSA + LPS + CCL4	Patent	Distilled water	1,3,4,5,8

SD: Sprague-Dawley; BSA: albumin from bovine serum; LPS: lipopolysaccharide; SEB: staphylococcal enterotoxin-B; CCL4: carbon tetrachloride; ig:irrigation; h: hour; NR: NO report; D: days; W: weeks; 1: 24-hour urinary protein quantity; 2: urinary red blood cell; 3: serum creatinine; 4: blood urea nitrogen; 5: albumin; 6: transforming growth factor-*β*; 7: MMP-9 or matrix metallopeptidase 9/matrix metalloproteinase tissue inhibitor 1; 8: IL-6 or interleukin-6; 9: TNF-*α* or tumor necrosis factor-*α*; 10: nephrin mRNA; 11: uric acid; 12: alanine aminotransferase; 13: aspartate aminotransferase; 14: cholesterol; 15: triglyceride; 16: nuclear factor kappa-B; 17: monocyte chemotactic protein 1; 18: intercellular cell adhesion molecule-1; 19: nephrin; 20: podocin; 21: podocin mRNA; 22: P38MAPK; 23: superoxide dismutase; 24: malondialdehyde; 25: interleukin-13 mRNA; 26: interleukin-4; 27: immunoglobulin A; 28: immunoglobulin G; 29: immunoglobulin M; 30: C3; 31: prothrombin time; 32: prothrombin time activity; 33: activated partial thromboplastin time; 34: thrombin time; 35: fibrinogen; 36: nitric oxide; 37: nitric oxide synthase.

**Table 2:** Quality assessment of included studies.

Study	A	B	C	D	E	F	G	H	I	J
Bian et al., 2016	U	U	U	U	U	U	U	U	Y	Y
Chen et al., 2013	Y	U	U	U	U	U	U	U	Y	Y
Guo et al., 2010	Y	U	U	U	U	U	U	U	Y	Y
Huang et al., 2009	U	U	U	U	U	U	U	Y	Y	Y
Jin et al., 2017	U	U	U	U	U	U	U	U	Y	Y
Liu et al., 2003	U	U	U	U	U	U	U	U	Y	Y
Song et al., 2014	Y	U	U	U	U	U	U	U	Y	Y
Tang et al., 2018	Y	U	U	U	U	U	U	U	Y	Y
Wang et al., 2018	U	U	U	U	U	U	U	U	Y	Y
Wang et al., 2010	U	U	U	U	U	U	U	Y	Y	Y
Wang and wang, 2017	U	U	U	U	U	U	U	U	Y	Y
Xiang et al., 2013	U	U	U	U	U	U	U	U	Y	Y
Yang et al., 2018	U	U	U	U	U	U	U	U	Y	Y
Yang et al., 2017	U	U	U	U	U	U	U	U	Y	Y
Zhang et al., 2016	U	U	U	U	U	U	U	U	Y	Y
Zhang, 2013	U	U	U	U	U	U	U	Y	Y	Y
Zhou et al., 2015	Y	U	U	U	U	U	U	U	Y	Y
Zou et al., 2016	U	U	U	U	U	U	U	Y	Y	Y
Zeng et al., 2004	U	U	U	U	U	U	U	U	Y	Y
Hu et al., 1999	U	U	U	U	U	U	U	U	Y	Y
Lin et al., 2016	U	U	U	U	U	U	U	U	Y	Y
Liu et al., 2006	U	U	U	U	U	U	U	Y	Y	Y
Sun et al., 2004	U	U	U	U	U	U	U	U	Y	Y
Wang et al., 2017	U	U	U	U	U	U	U	U	Y	Y
Yan et al., 2015	U	U	U	U	U	U	U	U	Y	Y
Ye et al., 2014	U	U	U	U	U	U	U	Y	Y	Y
Zhou et al., 2003	U	U	U	U	U	U	U	U	Y	Y
Cao et al., 2009	U	U	U	U	U	U	U	U	Y	Y
Yu et al., 2013	U	U	U	U	U	U	U	U	Y	Y
Zhang et al., 2022	U	U	U	U	U	U	U	Y	Y	Y

A, whether the distribution sequence is generated or applied sufficiently; B, whether the baselines of each group are the same; C, whether the distribution hiding is sufficient; D, whether the animals are randomly placed during the experiment; E, whether the researchers are blinded; F, whether the animals are randomly selected in the result evaluation; G, whether the blind method is used for the evaluators of the results; H, whether the incomplete data are reported; I, whether the research report has nothing to do with the selective results report; J, whether there is no other bias; Y, yes; N, no; U, uncertain.

## Data Availability

The datasets used and/or analyzed during the present study are available from the corresponding author upon request.
